# The safety and efficacy of escitalopram and sertraline in post-stroke depression: a randomized controlled trial

**DOI:** 10.1186/s12888-024-05833-w

**Published:** 2024-05-15

**Authors:** Ning Yan, Shaohua Hu

**Affiliations:** 1 Department of Psychiatry, the First Affiliated Hospital, Zhejiang University School of Medicine, Hangzhou, 310003 China; 2 Department of Psychiatry, Shanghai Jing’an District Mental Health Center, Shanghai, 200040 China; 3 The Key Laboratory of Mental Disorder’s Management in Zhejiang Province, Hangzhou, 310003 China; 4 Brain Research Institute of Zhejiang University, Hangzhou, 310003 China; 5 Zhejiang Engineering Center for Mathematical Mental Health, Hangzhou, 310003 China; 6 Department of Neurobiology, NHC and CAMS Key Laboratory of Medical Neurobiology, School of Brain Science and Brian Medicine, and MOE Frontier Science Center for Brain Science and Brain-Machine Integration, Zhejiang University School of Medicine, Hangzhou, 310003 China; 7 Department of Psychology and Behavioral Sciences, Zhejiang University, Hangzhou, 310003 China

**Keywords:** Escitalopram, Sertraline, Post-stroke depression, Anxiety, Overall response rate

## Abstract

**Objectives:**

This study aims to evaluate the safety and efficacy of escitalopram and sertraline in post-stroke depression (PSD) patients, to provide more reliable therapeutics for cardiovascular and psychiatric clinical practice.

**Methods:**

We recruited 60 patients (aged 40–89 years old) with an ICD-10 diagnosis of PSD, who were then randomly assigned to two groups and treated with flexible doses of escitalopram (10 to 20 mg/day, *n* = 30) or sertraline (50 to 200 mg/day, *n* = 30) for consecutive 8 weeks, respectively. The 24-item Hamilton Depression Rating Scale (HAMD-24), the 14-item Hamilton Anxiety Rating Scale (HAMA-14), the Treatment Emergent Symptom Scale (TESS), the Montreal Cognitive Assessment Scale (MOCA), and the Activity of Daily Living scale (ADL) were used to assess patients before, during, and after treatment for depression, anxiety, adverse effects, cognitive function, and daily living activities. Repeated measures ANOVA, the Mann–Whitney U test, the chi-square test (χ^2^), or Fisher’s exact test was employed to assess baseline demographics, response rate, adverse effects rate, and changes in other clinical variables.

**Results:**

Significant reduction in HAMD-24 and HAMA-14 scores was evaluated at baseline, as well as 1, 3, 4, 6, and 8 weeks of drug intervention (*p* < 0.01). There was a significant group difference in post-treatment HAMD-24 scores (*p* < 0.05), but no difference was observed in HAMA-14 scores (*p* > 0.05). Further analysis showed a significant variance in the HAMD-24 scores between the two groups at the end of the first week (*p* < 0.01). The incidence of adverse effects in both patient groups was mild, but there was a statistically significant difference between the two groups (*p* < 0.05). The improvement in cognitive function and the recovery of daily living abilities were comparable between both groups (*p* > 0.05).

**Conclusion:**

Escitalopram and sertraline showed comparable efficacy for anxiety symptoms, cognitive function, and daily living abilities in PSD patients. In addition, escitalopram was more appropriate for alleviating depressive symptoms. To validate the conclusion, trials with a larger sample size are in demand in the future. The registration number is ChiCTR1800017373.

## Introduction

Recently, a rapid-growing number of patients suffering from cardiovascular and cerebrovascular disease (CVD) has been witnessed, posing a significant threat to the health of the middle-aged and elderly population. Epidemiological data from both domestic and international reports consistently indicate that CVD remains the leading cause of mortality in the general population [1, 2]. Stroke is the primary subtype of CVD, resulting in various prevalent and detrimental complications including PSD [2, 3]. PSD can substantially impair the recovery of neurological and cognitive function, resulting in reduced quality of life [4–6]. Studies indicate that approximately 30% of stroke patients develop PSD within one year, and the mortality rate for those with PSD is significantly higher compared to those without PSD within ten months following the stroke onset [7–9]. Moreover, it has also been found that the occurrence of PSD remains high two years after the stroke attack, ranging from 11 to 41% [7].

A bidirectional association between depression and stroke has been noted. Stroke increases the risk of PSD, while depression acts as a distinct risk factor for stroke. Despite the acknowledged negative impact on social functional recovery and disease prognosis, PSD is consistently disregarded in clinical practice. Evidence highlights that early intervention of antidepressant therapies in stroke patients presenting no depression symptoms decreases the risk of developing depression [8]. Hence, it is crucial to provide safer and more effective medications to alleviate the health and financial burden caused by PSD.

Currently, the evidence of the preventive and therapeutic methods for PSD is insufficient. Previous studies have revealed that managing depression in patients with neurological disorders such as stroke faces greater difficulty compared to depression treatment among those without, probably because of the delayed diagnosis and intervention [9]. Consequently, challenges remain in treating patients suffering from PSD, which might eventually lead to suicide. Given the significant influence of PSD on families and society, more attention, early identification, and effective treatment are of great importance for improving the long-term prognosis of the patients.

Up to now, selective serotonin reuptake inhibitors (SSRIs) have been widely recognized as the preferred pharmacological intervention for PSD [10]. Previous investigations have consistently demonstrated that the timely administration of SSRIs not only substantially decreases the occurrence of PSD, but also comprehensively enhances motor function, neurological function, and social function recovery after stroke [11]. Evidence-based medicine treatment guidelines show that the efficacy and safety of escitalopram and sertraline are superior to other SSRIs, with sertraline being the preferred choice for patients with PSD due to fewer contraindications [12]. However, only about 60%-80% of patients with depressive disorders respond effectively to sertraline treatment in clinical practice, and there are also a significant number of patients who change medications due to intolerable adverse effects of sertraline. Therefore, it is necessary to explore other safer and more effective medications. In recent years, there has been increasing research on escitalopram, with growing evidence suggesting that compared to other SSRIs, escitalopram has advantages in improving emotions, motor function, cognitive function, and promoting immune function recovery in PSD patients, with faster onset, milder adverse effects, and is more suitable to treat PSD patients than sertraline [4–6, 13–16]. To validate the above conclusions, this study aims to further compare the efficacy and safety of escitalopram and sertraline in treating PSD, to provide patients with more effective, individual, and reliable therapies.

## Patients and methods

### Patients

This study was conducted at the Affiliated First Hospital of Zhejiang University School of Medicine, with a focus on recruiting middle-aged and elderly stroke patients. Sixty patients with PSD, including both inpatients and outpatients, were enrolled in the study from March^1st^ 2020 to April^30th^ 2022. The diagnosis was conducted using semi-structured questionnaires administered by two professionally trained psychiatrists. All patients were voluntary and had the option to withdraw at any time. The double-blind, randomized, and controlled trial received approval from the Ethics Committee of the Affiliated First Hospital of Zhejiang University School of Medicine. The registration number is ChiCTR1800017373 (registration date: 26/07/2018).

Inclusion Criteria: a. Diagnosed with PSD (ischemic) according to ICD-10, confirmed through magnetic resonance imaging examinations or computed tomography examinations, and who have experienced depressive symptoms after the onset of stroke, b. Age between 40–89 years old, c. Not recently treated with electroconvulsive therapy or repetitive transcranial magnetic stimulation, d. Have a permanent address and be able to complete a 2-month follow-up, e. Have duly endorsed the informed consent and have a comprehensive comprehension of the study content, f. Meet the initial positive criteria as determined by the Patient Health Questionnaire-9.

Exclusion criteria: a. Presence of other severe physical diseases including organic brain diseases, b. With a history of substance abuse, c. With comorbid psychiatric disorders, d. With suicidal ideation or behavior, e. Pregnancy, lactating, or within two months of pregnancy planning, f. With a history of individual or familial epilepsy, g. With mental retardation, h. Have participated in any clinical trials within the past 2 months. Furthermore, patients with laboratory tests three times higher than normal must seek the opinion of the investigator before enrollment.

### Methods

#### Study methods

Random tables and corresponding codes were generated by the Statistical Analysis System (SAS) to assign each patient an equal chance of being allocated to two different treatment groups. To ensure the objectivity of the study results, assessors were blinded to the group allocation of the patients when rating the scales. Demographic characteristics of the patients, including age, gender, race, disease course, laboratory test, electrocardiogram, and results of physical examination, were collected. The baseline assessment was conducted using the HAMD-24 and HAMA-14. In this trial, patients who fulfilled the inclusion criteria were randomly allocated, to either the escitalopram group (treatment group, TG; *n* = 30, H. Lundbeck A/S, batch number J20100165) or the sertraline group (control group, CG; *n *= 30, Pfizer Pharmaceuticals, batch number H10980141), in a 1:1 ratio. The TG was administered an initial daily dosage of 10 mg, which was incrementally titrated up to 20 mg within 2 weeks. The CG commenced with a daily dose of 50 mg, which was gradually increased to 100 mg within 2 weeks. The maximum dose for sertraline was 200 mg/day. All patients underwent an 8-week follow-up study. The HAMD-24 and HAMA-14 scores were assessed at baseline and at the end of 1, 3, 4, 6, and 8 weeks during the treatment. Adverse events including abnormal liver function, dizziness, fatigue, nausea, vomiting, insomnia, and constipation were recorded using the TESS. The MOCA and the ADL were used to assess patients before and after treatment for cognitive function and daily living activities. No additional or adjunctive antidepressant treatment was administered throughout the study. Power calculations showed that a sample size of 50 sufficed to achieve a power of 0.80 for detecting an effect size of 0.50 (at an alpha level of 0.05).

#### Efficacy and evaluation criteria

The efficacy of the treatment was determined based on a reduction rate of over 50% in HAMD-24, while meeting the criteria of a total HAMD-24 score of ≤ 8 is defined as clinical treatment remission. (To facilitate the statistical analysis, the scores of the HAMD-24: A reduction rate of ≥ 75% was considered as complete remission, a reduction rate of ≥ 50% but < 75% was considered as partial remission, a reduction rate of ≥ 25% but < 50% was considered as mild remission, and a reduction rate of < 25% was considered as no remission.)

#### Safety evaluation

Safety and tolerability assessments encompassed the determination of adverse event incidence and severity, which were consistently observed throughout the treatment duration. Comprehensive laboratory tests and electrocardiographic examinations were conducted on all patients prior to treatment and post-treatment (at 8 weeks). Two patients in both the TG and the CG withdrew from the trial due to intolerance to adverse events. (As assessed by clinical physicians, the aforementioned patients did not experience severe adverse effects, but withdrew from the trial due to subjective reasons rejecting further drug treatment). Overall, the safety profile of the study was favorable.

### Statistical analysis

This study utilized an intention-to-treat (ITT) analysis to assess the primary outcomes. The ITT analysis includes all randomized patients who have received at least one dose of medication, regardless of actual treatment compliance, subsequent withdrawal from treatment, or deviation from the treatment protocol, for statistical analysis purposes. All statistical tests were performed using the Statistical Package for the Social Sciences software v. 25.0 (IBM Corp., Armonk, NY., USA). Non-normally distributed data are presented as median (M), 25th percentile (P25), and 75th percentile (P75). Normally distributed data are presented as mean ± standard deviation (SD). Independent samples T-test was employed for continuous variables with a normal distribution, whereas the Mann–Whitney U test was used for non-normally distributed data. Categorical variables were compared using the chi-square test (χ^2^) or Fisher’s exact test and expressed as percentages. The aforementioned approach was employed to assess baseline demographics, response rate, adverse effects rate, and other clinical variables changes. The reduction in HAMA-14 and HAMD-24 scores between groups was compared using repeated measures ANOVA. A *p*-value < 0.05 was considered statistically significant (two-tailed).

## Results

### Demographic information

A total of 60 patients diagnosed with PSD, including both outpatients and inpatients, were consecutively enrolled in this clinical trial. Among them, 30 patients were allocated to the TG and 30 patients to the CG. Two patients in the TG withdrew from the trial voluntarily due to intolerable adverse effects at the end of the 1st and 4th follow-up weeks. Two patients in the CG withdrew from the trial at the 2nd and 6th weeks due to intolerable adverse effects and poor drug treatment efficacy, while the remainder completed the 2-month follow-up. No significant differences were observed between the two groups in the disease course, age, or other demographic characteristics (*p* > 0.05) (Table 1).

**Table 1:** Demographic characteristics

Characteristic	TG (*n* = 30)	CG (*n* = 30)	*P*-value*
Gender (n, male/female)	8/22	8/22	1.000
Age (years)	67.0 (57.5, 72.3)	66.5 (56.0, 71.3)	0.657
Educational background (years)	6.0 (0.0, 9.0)	7.5 (0.0, 12.0)	0.178
ADL (score)	19.5(18.00,21.25)	19.0(18.00,22.00)	0.964
Moca (score)	18.5 (14.3, 23.3)	20.5 (15.6, 24.0)	0.423
Total disease course (mouth)	10.0 (8.0, 11.0)	10.0 (8.0, 12.0)	0.839
Heart disease (n, %)	7.0 (23.3%)	9.0 (30%)	0.559
Hyperlipidemia (n, %)	1.0 (3.3%)	1.0 (3.3%)	1.000
Carotid plaque (n, %)	3.0 (10%)	4.0 (13.3%)	1.000
Hypertension (n, %)	11.0 (36.7%)	17.0(56.7%)	0.121
Baseline HAMD-24 (score)	26.68** ± **6.56	28.30** ± **4.98	0.177
Baseline HAMA-14 (score)	20.07** ± **4.57	21.70** ± **4.03	0.087

*TG* treatment group, *CG* control group, *HAMA-14* 14-item Hamilton Anxiety Rating Scale, *HAMD-24* 24-item Hamilton Depression Rating Scale, *MOCA* Montreal Cognitive Assessment Scale, *ADL* Activity of Daily Living. Data were expressed as mean ± SD or M (P25, P75)

*Significant at *P* < 0.05 (two-tailed)

### Comparison of HAMD-24 scores between the TG and CG

**Fig. 1:**
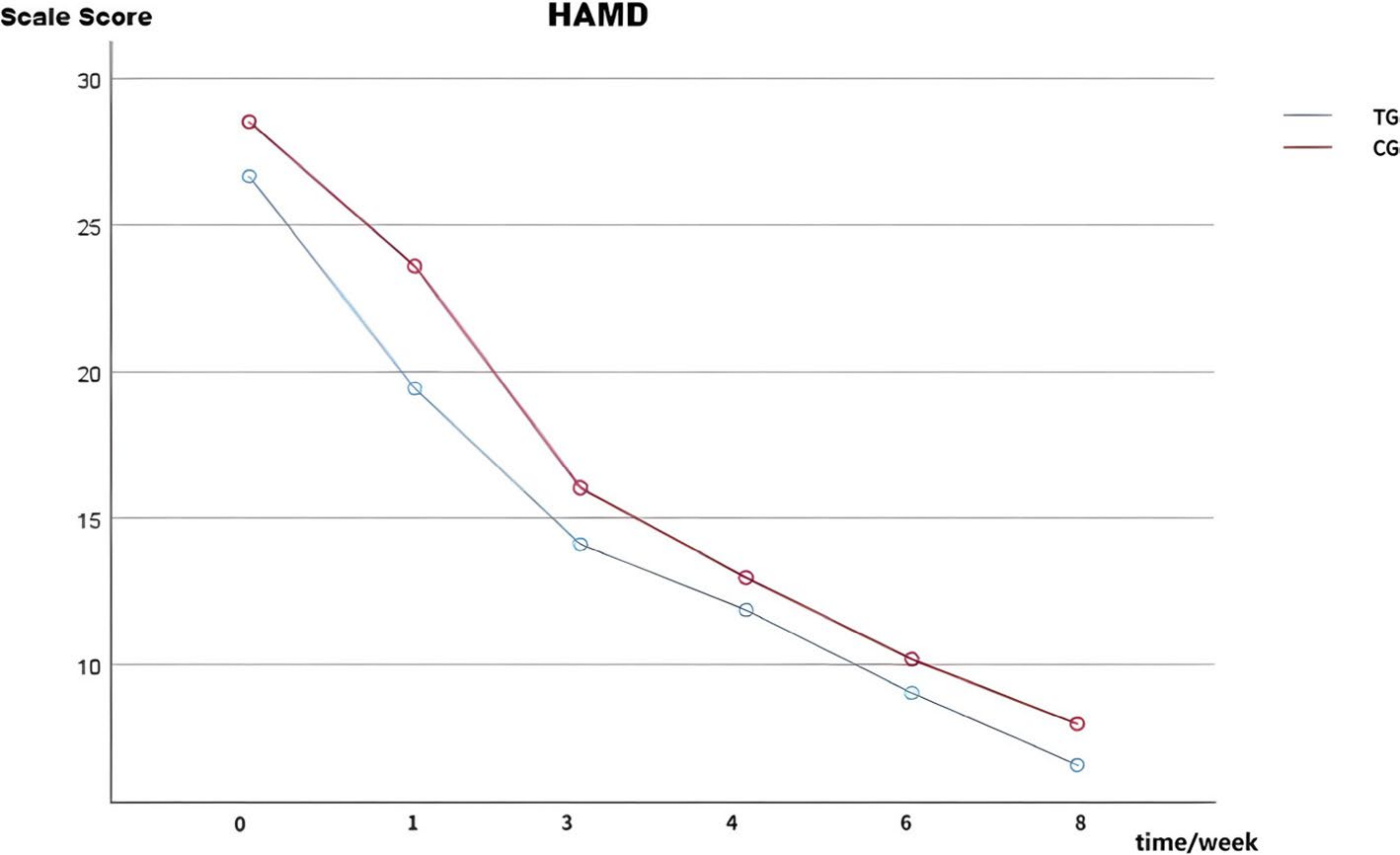
Changes in HAMD-24 scores of the escitalopram and sertraline group during the follow-up (*n* = 30) The HAMD-24 scores exhibited a consistent decrease at 1, 3, 4, 6, and 8 weeks post-treatment (*P* < 0.05), with a statistically significant difference observed between the TG and CG (*p* < 0.05). At the conclusion of the initial week, there was a significant difference in the comparison of HAMD-24 scores between the TG and CG (*p* < 0.05). Abbreviations: TG: treatment group; CG: control group; HAMD-24: 24-item Hamilton Depression Rating Scale

Prior to treatment, no statistically significant difference was observed (T = 1.366, *p* > 0.05). However, one week after treatment initiation, a significant decrease in HAMD-24 scores was observed, with a statistically significant difference in both the TG and CG (T = 2.620, *p* < 0.05). This period exhibited the most rapid rate of decline. Subsequently, at 1, 3, 4, 6, and 8 weeks after treatment, the HAMD-24 scores continued to decrease, with a statistically significant difference in both the TG and CG (*F* = 4.068,* p* < 0.05) (See Table 2 and Fig. 1).

**Table 2:** Comparison of HAMD-24 scores between the TG and CG

Items	TG (*N* = 30)	CG (*N* = 30)	*P*-value*	TIME	Group^②^	TIME*Group
HAMD baseline	26.68 ± 6.56	28.30 ± 4.98				
HAMD 1 week	19.43 ± 6.79^①^	23.61 ± 6.01^①^	*p* < 0.001	*F* = 258.618	*F* = 4.068	*F* = 1.245
HAMD 3 week	14.11 ± 5.97	16.04 ± 4.87	*p* < 0.001	*p* < 0.001	*p* = 0.049	*p* = 0.294
HAMD 4 week	11.86 ± 5.37	12.96 ± 4.84	*p* < 0.001			
HAMD 6 week	9.04 ± 5.03	10.18 ± 4.51	*p* < 0.001			
HAMD 8 week	6.57 ± 4.26	7.96 ± 3.83	*p* < 0.001			

Repeated measures ANOVA was conducted. *Represents the comparison of HAMD-24 scores at 1, 3, 4, 6, and 8 weeks with baseline between the two groups. Significant at *P* < 0.05 (two-tailed); ^①^Significant differences were observed in the comparison of HAMD-24 scale scores between the TG and CG one week after treatment initiation (*P* < 0.05). ^②^Represents the comparison of differences between the two groups. *TG* treatment group, *CG* control group, *HAMD-24* 24-item Hamilton Depression Rating Scale. Data were expressed as mean ± SD

### Comparison of HAMA-14 scores between the TG and CG

**Fig. 2:**
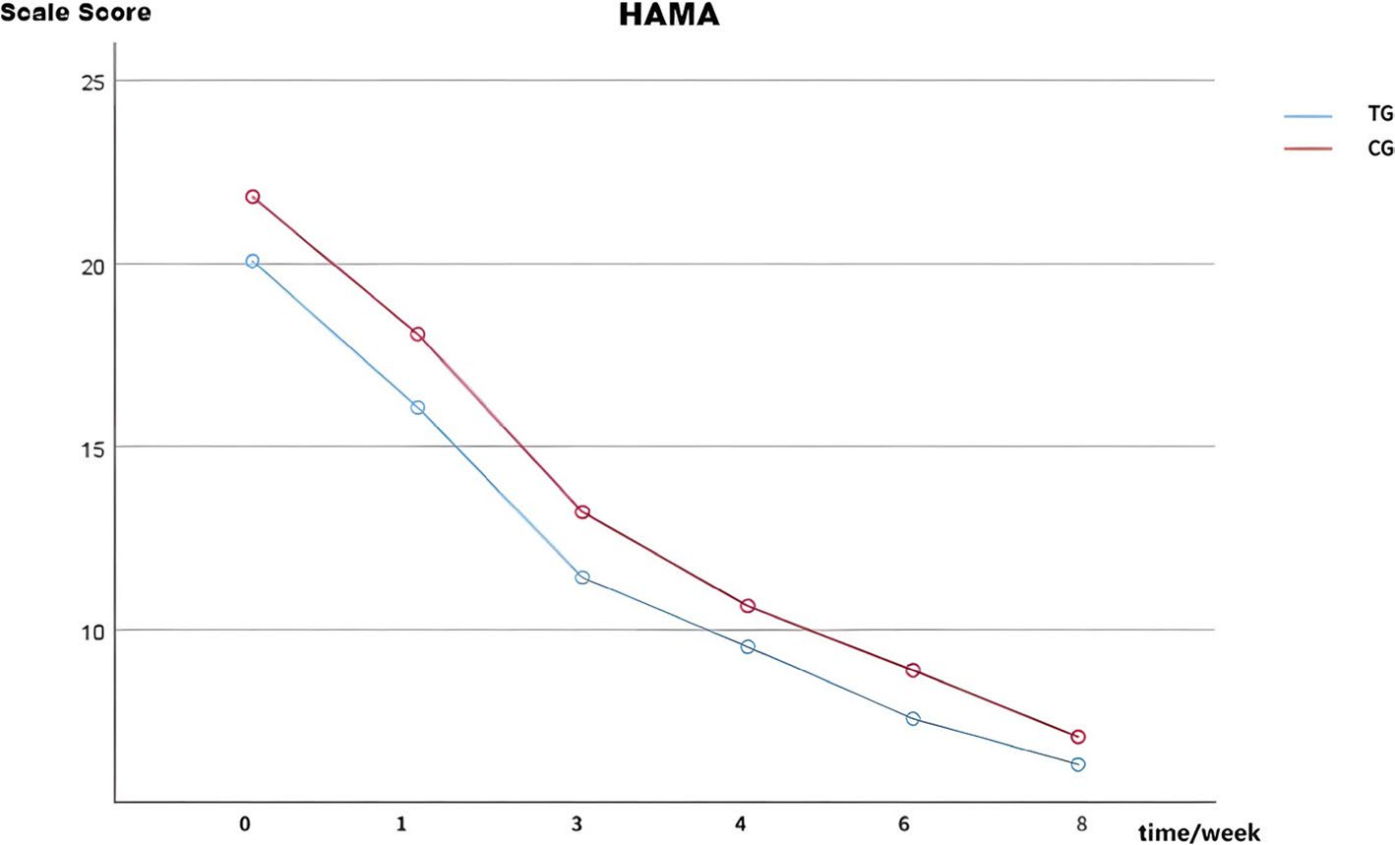
Changes in HAMA-14 scores of the escitalopram and sertraline group during the follow-up (*n* = 30). The HAMA-14 scores exhibited a consistent decrease at 1, 3, 4, 6, and 8 weeks post-treatment (*P* < 0.05). No statistically significant difference was observed in both groups (*p* > 0.05). Abbreviations: TG: treatment group; CG: control group; HAMA-14: 14-item Hamilton Anxiety Rating Scale

Prior to treatment, no statistically significant difference was observed (T = 1.740, *p* > 0.05). Subsequently, at 1, 3, 4, 6, and 8 weeks after treatment, the HAMA-14 scores continued to decrease, with no statistically significant difference in both the TG and CG (*F* = 3.185, *p* > 0.05) (See Table 3 and Fig. 2).

**Table 3:** Comparison of HAMA-14 scores between the TG and CG

Items	TG (*N* = 30)	CG (*N* = 30)	*P*-value*	TIME	Group^①^	TIME*Group
HAMA baseline	20.07 ± 4.57	21.70 ± 4.03				
HAMA 1 week	16.07 ± 4.71	18.07 ± 4.70	*p* < 0.001	*F* = 238.388	*F* = 3.185	*F* = 0.351
HAMA 3 week	11.43 ± 4.13	13.21 ± 3.60	*p* < 0.001	*p* < 0.001	*p* = 0.080	*p* = 0.759
HAMA 4 week	9.54 ± 3.59	10.64 ± 3.75	*p* < 0.001			
HAMA 6 week	7.57 ± 3.83	8.89 ± 3.93	*p* < 0.001			
HAMA 8 week	6.32 ± 3.22	7.07 ± 3.38	*p* < 0.001			

Repeated measures ANOVA was conducted. *Represents the comparison of HAMA-14 scores at 1, 3, 4, 6, and 8 weeks with baseline between the two groups. Significant at *P* < 0.05 (two-tailed); ^①^Represents the comparison of differences between the two groups. *TG* treatment group, *CG* control group, *HAMA-14* 14-item Hamilton Anxiety Rating Scale. Data were expressed as mean ± SD

### Comparison of MOCA scores between the TG and CG

After 8 weeks of treatment, there was no statistically significant difference in cognitive function improvement between the two groups (Z = 1.040, *p* > 0.05) (Table 4).

**Table 4:** Comparison of MOCA scores between the TG and CG (score)

Group	MOCA baseline	MOCA 8 week	U test	
			Z	*P*-value
TG (*N* = 30)	18.5(14.25,23.25)	21.5(17.25,26.00)	4.601	*P* < 0.001
CG (*N* = 30)	20.5(15.75,24.00)	24.0(18.75,26.00)	4.527	*P* < 0.001
Z	0.801	1.040		
*P*-value	0.423	0.298		

*TG* treatment group, *CG* control group, *MOCA* Montreal Cognitive Assessment

^*^Significant at *P* < 0.05 (two-tailed)

### Comparison of ADL scores between the TG and CG

After 8 weeks of treatment, both groups of patients showed improvement in activities of daily living, with similar levels of recovery observed in the two groups (Z = 0.143, *p* > 0.05) (Table 5).

**Table 5:** Comparison of ADL scores between the TG and CG (score)

Group	ADL baseline	ADL 8 week	U test	
			Z	*P*-value
TG (*N* = 30)	19.5(18.00,21.25)	16.0(14.75,18.25)	4.676	*P* < 0.001
CG (*N* = 30)	19.0(18.00,22.00)	16.0(15.00,18.00)	4.719	*P* < 0.001
Z	0.045	0.143		
*P*-value	0.964	0.886		

*TG* treatment group, *CG* control group, *ADL* Activity of Daily Living

^*^Significant at *P* < 0.05 (two-tailed)

### Clinical efficacy

The overall response rate in both groups was comparable, and the observed difference was not statistically significant. (χ^2^ = 3.042, *p* > 0.05) (Table 6).

**Table 6:** Comparison of clinical efficacy between the TG and CG (n, %)

Groups	N	Complete remission	Partial remission	Mild remission	No remission	Overall response rate	χ^2^ value	*P*-value*
TG	30	19 (63.3)	7 (23.3)	2 (6.7)	2 (6.7)	86.67	3.042	0.408
CG	30	14 (46.7)	13 (43.3)	1 (3.3)	2 (6.7)	90.0		

*TG* treatment group, *CG* control group

^*^Significant at *P* < 0.05 (two-tailed)

### Adverse effects

No serious adverse events were observed among all patients. The adverse effects in both groups were comparable, and there was a statistically significant observation (χ^2^ = 9.097, *p* < 0.05) (Table 7).

**Table 7:** Comparison of adverse effects between the TG and CG (n, %)

Groups	N	Gastrointestinal reaction	Dizziness	Abnormal liver function	Insomnia	Constipation	χ^2^ value	*P*-value*
TG	30	3 (10)	1 (3.3)	1 (3.3)	1 (3.3)	0 (0.0)	9.097	0.046
CG	30	7 (23.3)	5 (16.7)	0 (0.0)	0 (0.0)	2 (6.7)		

*TG* treatment group, *CG* control group

^*^Significant at *P* < 0.05 (two-tailed)

## Discussion

The randomized controlled study aimed to investigate the safety and efficacy of escitalopram and sertraline in treating PSD. The main findings showed significant improvement in the clinical symptoms of both escitalopram and sertraline. Both groups had similar effects in alleviating anxiety (*F* = 3.185, *p* > 0.05), improving cognitive function (Z = 1.040, *p* > 0.05), and recovering daily living abilities (Z = 0.143, *p* > 0.05). However, escitalopram showed superior efficacy in alleviating depression symptoms compared to sertraline (*F* = 4.068, *p* < 0.05). Further analysis showed a quicker onset of effect for escitalopram at the one week after treatment initiation (T = 2.620, *p* < 0.05). Furthermore, the overall response rate showed no significance between the TC and CG, while the incidence of adverse effects was lower in patients receiving escitalopram intervention. We can conclude that escitalopram should be recommended for PSD treatment. Although larger cohorts with strict blinding and randomization are needed for further exploration and deeper insights into the specific safety and efficacy of escitalopram, the findings of our study still deserve increased attention.

At present, evidence-based pharmacotherapy for PSD remains insufficient. Given the chronic and recurrent disease course of PSD, long-term treatment is frequently required. Consequently, ensuring the tolerability and safety of medications becomes a crucial factor to consider. Prior investigations have demonstrated that escitalopram is the most effective and advantageous antidepressant for patients with PSD [17]. It has shown excellent efficacy in both acute and remission treatment phases [18]. Our study also arrived at a similar conclusion, suggesting that escitalopram offers superior efficacy and safety for PSD. Furthermore, compared to sertraline, escitalopram has a lower incidence of adverse events, which is consistent with our research findings. Particularly, a study reported that a fixed dose of 10 mg/day escitalopram has comparable clinical efficacy in treating depression symptoms to flexible-dose sertraline (50–200 mg/day) [15]. Both drugs are well-tolerated at these doses, indicating that low-dose escitalopram is generally safe and effective. It is worth noting that although increasing the dose of sertraline is associated with improved efficacy, the risk of adverse effects significantly increases when the doses above 150 mg/day [19]. So, when choosing the optimal dosage of sertraline, we need to consider the dose-dependence of both safety and efficacy.

Escitalopram and sertraline exert their antidepressant effects by inhibiting the reuptake of 5-HT at the presynaptic membrane, thereby elevating the concentration of 5-HT in the synaptic cleft [14, 15, 20]. Meanwhile, they exhibit a remarkably high affinity for the sodium-dependent serotonin transporter protein (SERT). The SERT is a primary target responsible for the therapeutic actions of these medications. Among SSRIs, escitalopram, the S-enantiomer of citalopram, demonstrates the utmost selectivity, being at least 60-fold more selective than sertraline [14, 21]. The SERT has one or more allosteric sites. Compared to sertraline, escitalopram exhibits the ability to simultaneously bind to both the allosteric sites and orthosteric sites, demonstrating its exceptional selectivity for SERT [14, 15, 20]. Escitalopram's binding to SERT's allosteric site not only enhanced its binding to the orthosteric site but also blocked its dissociation from the orthosteric site [20, 22, 23]. Escitalopram is the sole antidepressant targeting SERT that exhibits both chiral advantages and dual allosteric [14]. Consequently, it demonstrates a faster onset of action and higher efficacy compared to sertraline, which is consistent with our research findings. In recent years, numerous scientific studies by other scholars have confirmed this conclusion. Sanchez et al. propose that escitalopram is the preferred medication, exhibiting superior efficacy and tolerability compared to paroxetine and sertraline [14]. Li et al. suggest that among 9 types of antidepressants, escitalopram demonstrates the best efficacy for PSD [17]. Kalbouneh et al. argue that escitalopram offers the most stable therapeutic effect for PSD [16].

Escitalopram has little inhibitory action on other receptors, including the cholinergic muscarinic M1, adrenergic α, histamine H1, adrenergic β, and dopamine receptors [14, 15, 20, 21, 23]. This characteristic results in relatively fewer adverse effects and minimal impact on patient weight. Furthermore, escitalopram has a negligible impact on hepatic cytochrome P450 enzyme metabolism in liver cells, and long-term use does not lead to drug accumulation in the body. It also presents no significant contraindications regarding drug compatibility [20, 23]. It is worth mentioning that sertraline exhibits a substantial inhibitory effect on specific cytochrome P450 enzymes, potentially leading to a higher likelihood of drug-drug interactions compared to escitalopram [14]. In alignment with our study results, no severe adverse events were observed with both medications, and escitalopram demonstrated a higher safety profile than sertraline. Feng et al.'s latest meta-analysis also confirms that escitalopram is safer for PSD, effectively improving not only depressive symptoms but also motor function. For stroke patients in the rehabilitation stage, early treatment with escitalopram can improve neural functional prognoses and endothelial dysfunction [4, 11, 24]. Consequently, escitalopram significantly facilitates the recovery of cognitive function and neurological dysfunction, ultimately reducing disability rates and improving patients’ quality of life. Regrettably, we did not assess any improvement in neurological dysfunction before and after treatment, only evaluating the enhancement in cognitive function and daily living activities. Future investigations should focus on assessing the recovery of neurological dysfunction in PSD pre- and post-treatment to acquire a more comprehensive comprehension of the potential mechanisms of escitalopram in the brain.

Through the aforementioned discussion, we have elucidated the advantages and efficacy of escitalopram. Moving forward from clinical practice and integrating previous research findings, we further discuss the rationale for prioritizing escitalopram in patients with PSD. Severe strokes induce brain inflammation, consequently upregulating inflammatory cytokines responsible [9]. The increased inflammatory cytokines exacerbate glutamate excitotoxicity, leading to the expansion of neuronal apoptosis and infarctions [6, 9]. Prior investigations have demonstrated that escitalopram may exert a protective effect on the brain by modulating anti-inflammatory mediators, facilitating the regeneration of hippocampal neurons, and enhancing cerebral blood circulation to ischemic brain tissues [11]. Escitalopram effectively inhibited the elevation of pro-inflammatory cytokine, and tumor necrosis factor-alpha in the serum. Moreover, escitalopram demonstrated a notable elevation in the serum concentrations of the anti-inflammatory cytokine interleukin-10 [25]. However, it has been reported that escitalopram induced a notable increase in the interleukin-6 and tumor necrosis factor-alpha levels in mammalian macrophage cells [26]. Hence, further comprehensive investigations are warranted to clarify the impact of escitalopram on the immune system. Regrettably, we did not record the plasma cytokines, so it cannot be determined whether the effects of the escitalopram were specially targeted to the inflammatory cytokine. In future studies, the determination of inflammatory cytokine should be added for more convincing conclusions.

In addition, escitalopram augmented the speed of emotional processing and strengthened the connectivity between the left amygdala and right angular gyrus, as well as the right amygdala and bilateral ventromedial prefrontal cortex during emotion processing [27]. Prior investigations have suggested that escitalopram may modulate the activity of the hypothalamic–pituitary–adrenal axis and exhibit a correlation with the therapeutic outcomes in the management of depression [11]. Escitalopram can modulate multiple targets beyond the 5-HT system. Recent research has indicated its effects on the salt-inducible kinase 1 -CREB-regulated transcription co-activator 1 system within the paraventricular nucleus [28]. The involvement of this system in the antidepressant mechanism of escitalopram has been observed. Additionally, escitalopram enhances the levels of innate immunity modulators and promotes a shift towards T helper 2 responses, thereby improving the function and abundance of T regulatory cells [29]. These findings highlight the promising clinical potential and applicability of early escitalopram intervention in the management of PSD. Lastly, a comprehensive risk–benefit analysis should consider the potential therapeutic effects, adverse events, and risks associated with the specific PSD condition being treated when prescribing SSRIs for PSD.

To date, SSRIs have been widely used for the prevention and treatment of PSD, but their long-term benefits remain inconclusive. Reports suggest potential risks of bleeding and fractures associated with SSRIs, leading to increased mortality and disability rates in PSD [30, 31]. Studies indicate that SSRIs may disrupt platelet function, potentially increasing hematoma volume and the risk of recurrent hemorrhagic stroke [32]. Additionally, research shows an elevated risk of fractures with SSRI use. PSD patients typically experience fractures within 6 months [31, 33]. Our study did not observe fractures or recurrent intracerebral hemorrhage in PSD over a relatively short treatment period, suggesting caution regarding the long-term safety of SSRI use in PSD. Therefore, when considering prolonged SSRI therapy for PSD, the potential risks of fractures and recurrent hemorrhagic stroke should be balanced against the benefits of improving depressive symptoms.

This study has several limitations. Firstly, the statistical power of the analyses was constrained by the limited sample size. However, it is worth noting that the enrolled patients received no additional treatment apart from the aforementioned drugs. Therefore, the findings of this study remain credible. Secondly, the period of follow-up in this trial was relatively short. The evaluation of long-term treatment outcomes was not conducted, which could potentially impact our overall conclusion. To reproduce these findings and gain a deeper understanding of the potential mechanism, it is imperative to carry out additional multi-center studies in the future, incorporating larger sample sizes and longer-term observations.

## Conclusion

Collectively, the results of this study offer preliminary evidence indicating the effective improvement of depression symptoms, anxiety symptoms, cognitive function, and activities of daily living abilities in PSD through the use of both escitalopram and sertraline. However, escitalopram may be superior to sertraline in improving depressive symptoms, demonstrating a rapid onset, favorable efficacy, and fewer adverse effects. Therefore, it is worthy of priority clinical application in the treatment of PSD. Further studies with improved designs are necessary to validate our findings.

## Data Availability

The datasets generated during and/or analyzed during the current study are not publicly available but are available from the first author on reasonable request.

## Abbreviations

PSD: Post Stroke Depression
CVD: Cardiovascular and Cerebrovascular Disease
SSRIs: Selective Serotonin Reuptake Inhibitors
CG: Control Group
TG: Treatment Group
SERT: Serotonin Transporter
SD: Standard Deviation
HAMD-24: 24-Item Hamilton Depression Rating Scale
HAMA-14: 14-Item Hamilton Anxiety Rating Scale
TESS: Treatment Emergent Symptom Scale
MOCA: Montreal Cognitive Assessment Scale
ADL: Activity of Daily Living scale
SAS: Statistical Analysis System
ITT: Intention-to-treat Analysis
